# Induction of ovulation in *Xenopus *without hCG injection: the effect of adding steroids into the aquatic environment

**DOI:** 10.1186/1477-7827-9-11

**Published:** 2011-01-21

**Authors:** Aoi Ogawa, Junpei Dake, Yu-ki Iwashina, Toshinobu Tokumoto

**Affiliations:** 1 Department of Biology, Faculty of Science, National University Corporation Shizuoka University, Shizuoka 422-8529, Japan

## Abstract

**Background:**

The African clawed frog, *Xenopus laevis*, is widely used in studies of oogenesis, meiotic cell cycle and early embryonic development. However, in order to perform such studies, eggs are normally collected after the injection of hCG into the dorsal lymph sac of fully-grown female frogs following pre-injection of PMSF. Although this protocol is established and used as standard laboratory approach, there are some concerns over whether the injections could cause the transmission of deleterious microorganisms. Moreover, these injection protocols require a competent skilled worker to carry out the procedure efficiently.

**Methods:**

Recently, we established a novel method to induce fish ovulation by simply adding the natural maturation-inducing hormone of teleosts, 17 alpha, 20 beta-dihydroxy-4-pregnen-3-one (17,20 beta-DHP), into the surrounding water. In the present study, we demonstrate how we can induce ovulation in frogs using the same methodology.

**Results:**

In frogs, progesterone was effective in the induction of oocyte maturation *in vitro*. We then examined the ability of progesterone to induce ovulation in frogs. However treatment of frogs with progesterone alone only occasionally induced ovulation *in vivo*. The number of oocytes and the frequency of ovulation were significantly lower than that induced by hCG-injection. Thus, conditions were improved by using a combination of progesterone with estradiol and by pre-treating frogs with low concentrations of progesterone or estradiol. Finally, we established an efficient means of inducing ovulation in frogs which involved pre-treatment of frogs with salt solution followed by a mixture of estradiol and progesterone at high concentration. The frequency and numbers of oocytes obtained were identical to those resulting from PMSG-hCG induction. Fertilization rate of eggs ovulated by the new treatment method was comparable to eggs obtained by hCG-injection and juveniles developed normally.

**Conclusions:**

To conclude, we have successfully developed a novel method to induce ovulation in frogs but without the need for a potentially harmful injection strategy.

## Background

*In vitro *maturation of oocytes in frogs has been shown to be induced by several types of steroid hormones [1]. The most potent steroid with inducing-activity is progesterone which triggers activation of the M-phase-promoting factor (MPF) thereby permitting oocytes to proceed in the meiotic cell cycle. Recently, a new class of membrane-bound progestin receptors (mPR) have been identified in fish [2,3]. An orthologue of mPR was subsequently discovered in *Xenopus *and demonstrated to mediate progesterone signals into the oocyte [4]. During the course of maturation, oocytes undergo significant morphological changes associated with progression of the meiotic cell cycle which includes breakdown of the oocyte nuclear envelope (germinal vesicle breakdown, GVBD). Matured oocytes are then extruded from the surrounding follicle cells (ovulation) to be spawned into the aquatic environment. Ovulated oocytes are spawned after a layer of jelly has been added during passage along the oviduct. Spawned *Xenopus *eggs have been widely used in research via the preparation of egg extracts for cell cycle studies, or in the analysis of early development mechanisms following artificial insemination *in vitro*.

Frog ovulation can be induced by hCG-injection either *in vitro *by administering the hormone directly to follicle-enclosed oocytes, or by injection of the hormone solution into the dorsal lymph sac [5].

Recently we established a method to induce fish ovulation *in vivo *by simply administering maturation-inducing steroid into the water (Tokumoto et al submitted). We also described a method to induce oocyte maturation in fish by treatment with an endocrine-disrupting chemical, diethylstilbestrol (DES) [6], a non-steroidal estrogen. We also described how oocyte maturation in fish could be prevented by pentachlorophenol (PCP) [7], a widely used biocide. Subsequently, we have demonstrated that the agonistic effect of DES occurs via the mPR by showing direct interactions between DES and mPR by steroid binding assays using recombinant protein expressed in cultured cells [8]. Our results clearly indicated that the membrane steroid receptor represents a potential novel target for endocrine disrupting chemicals (EDCs). In order to address the effects of EDCs *in vivo*, the effects of externally applied EDCs or steroid hormones were examined by simply adding the agents into water. Results showed that externally applied EDCs, as well as MIS, could induce or prevent oocyte maturation and ovulation in living fish.

In the present study we investigated the efficacy of applying steroids to the external aquatic environment upon the induction of ovulation in the aquatic frog, *Xenopus*. Our results demonstrated that ovulation in frogs can be induced by external steroids. This protocol is relatively simple and most importantly, avoids the use of injection needles.

## Methods

### Materials

Frogs (*Xenopus laevis*) were purchased from Jo-hoku Seibutsu Kyozai (Shizuoka, Japan). Autogenic frogs were also used. Frogs were treated following procedures approved by the Shizuoka University Animal Care Committee. Steroid hormones (Androstenedione, Progesterone, 17β-estradiol, Testosterone, 17,20β-DHP) were purchased from Sigma Chemical Co. (St. Louis, MO). [1, 2, 6, 7- ^3^H]-Progesterone (94 Ci/mmol) and [2, 4, 6, 7- ^3^H]-Estradiol-17β (84 Ci/mmol) were purchased from Amersham Pharmacia Biotechnology (Piscataway, NJ).

### Induction of ovulation by hormonal injection

Spawning was induced in sexually mature *Xenopus *females by injecting 100 I.U. pregnant mare serum gonadotropin (PMSG) followed 24 to 30 hrs later by the injection of 500 I. U. human chorionic gonadotropin (hCG). Females were transferred into a glass bottle with screw cap of 12 cm in diameter which contained 1 L of 0.1 M NaCl per frog. Frogs remained in the bottle at 20°C until ovulation was completed (normally 24 hrs).

### Induction of ovulation by steroids

Females were transferred into a same glass bottle used for hormonal injection which contained 1 L of 0.1 M NaCl per frog. For pre-treatment, females were exposed to steroids *in vivo *by adding steroids directly into the water at 20°C (from a 10,000-fold stock in ethanol). After incubation for 24 to 30 hrs, agents to induce ovulation were added and the frogs maintained at 20°C until ovulation was completed (normally 24 hrs).

### Determining the number of ovulated eggs

Ovulated eggs were collected into plastic dishes of 150 mm diameter and photographed by digital camera. Images were digitized and eggs visible on the images were scored using ImageJ software.

### Estimation of incorporation of steroids by ^3^H-labeled tracers

[^3^H]-Progesterone (^3^H-P) and [^3^H]-Estradiol-17β (^3^H-E) were added to stock solution of each steroid at 0.2 μM and 0.1 μM. To estimate the solubility of steroids in this experimental conditions, 100 μl of ^3^H-P containing 200 mM progesterone in ethanol added into 1 L of 0.1 M NaCl with 100 μl of 100 mM estradiol without ^3^H-E or 100 μl of ^3^H-E containing 100 mM estradiol in ethanol added into 1 L of 0.1 M NaCl with 100 μl of 200 mM progesterone without ^3^H-P. To estimate incorporation of steroids into frog tissues, frogs maintained in the bottle for 24 hrs. After anesthetized with Methyl *p*-Hydroxybenzoate, 0.1 g of blood, ovary and skeletal muscle from frogs were obtained. The radioactivity in the tissues was measured with a liquid scintillation counter (ALOKA LSC-6100).

### Fertilization

The fertilizing ability of eggs was assessed by *in vitro *insemination following methodology described previously [9]. Fertilization rate (in %) was calculated by determining the proportion of embryos developing to and beyond the 4-cell stage.

## Results

### Externally applied progesterone induces ovulation *in vivo*

**Figure 1:**
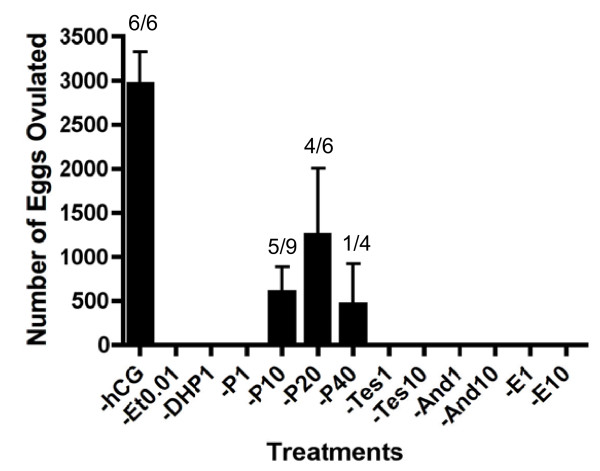
**The effect of various steroid hormones on the *in vivo *induction of *Xenopus *ovulation**. The left column represents the results of induction by hCG injection. Annotations underneath each column indicate substance and concentration for the induction of ovulation as following: Et0.01, ethanol 0.01%; DHP, 17,20β-DHP; P, progesterone; Tes, testosterone; And, androstenedion; E, 17β-estradiol. Each compound was added to 0.1 M NaCl at a final concentration of 1 μM except progesterone (additional high concentrations were examined at 10 μM; P10, 20 μM; P20, 40 μM; P40). After 24 to 30 hr incubation, the numbers of ovulated eggs were counted as described in the Materials and Methods. Each value represents the mean of data from more than three different females. The number of ovulated frogs per number treated is indicated over each column. Vertical lines indicate standard deviation.

**Figure 2:**
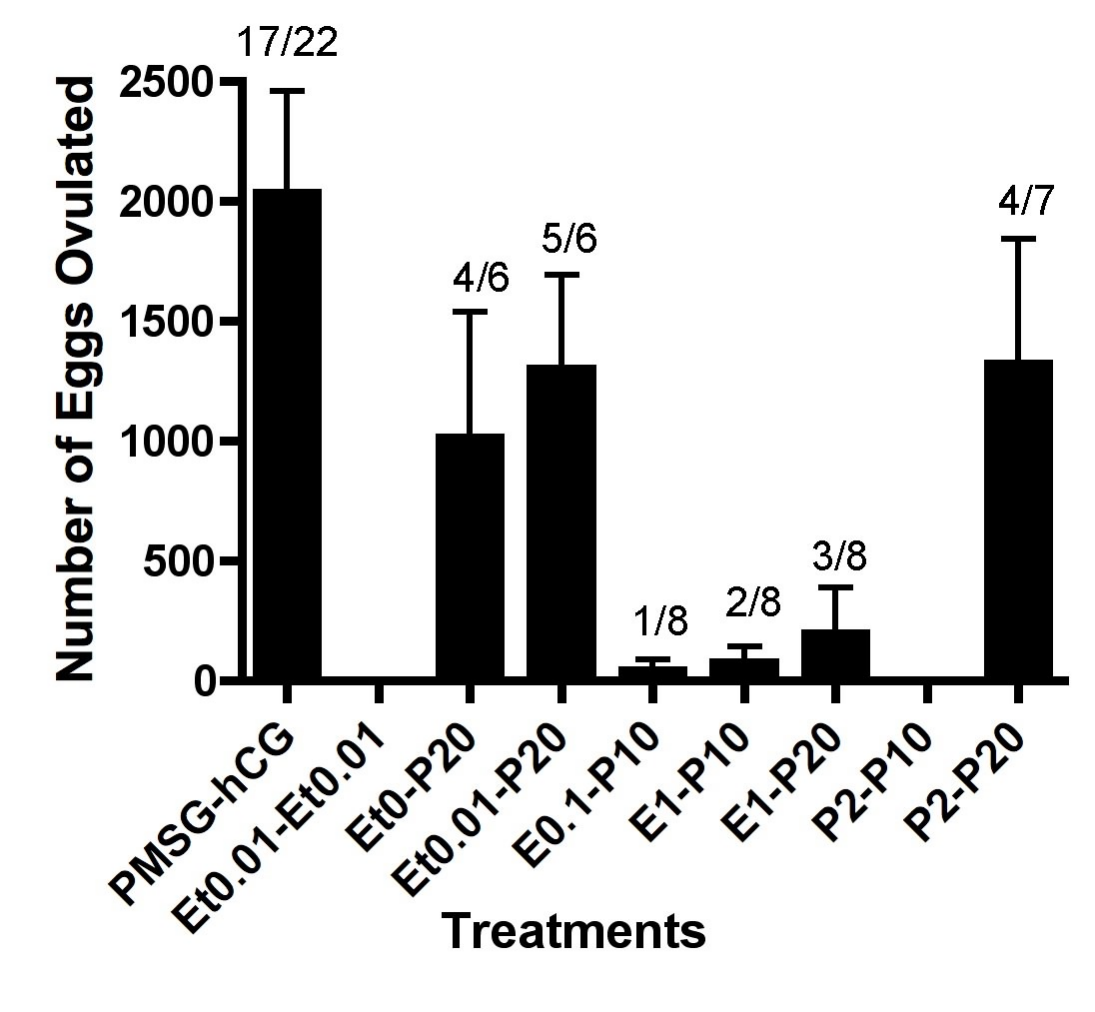
**The effect of steroid pre-treatment upon progesterone-induced ovulation**. Frogs were pre-treated with ethanol (Et), estradiol (E) or progesterone (P) at the indicated concentration for a period of one day before induction by progesterone. The left column represents induction results in response to PMSG-hCG injection. After 24 to 30 hr incubation, the number of ovulated eggs was counted as described in the Materials and Methods. Annotations underneath each column indicate substance and concentration for both pre-treatment and the induction of ovulation. For example, Et0-P20 represents pre-treatment with 0% ethanol for one day followed by induction by a mixture of 20 μM progesterone the following day. Each value represents the mean of data from more than three different females. The number of ovulated frogs per number treated is indicated over each column. Vertical lines indicate standard deviation.

**Figure 3:**
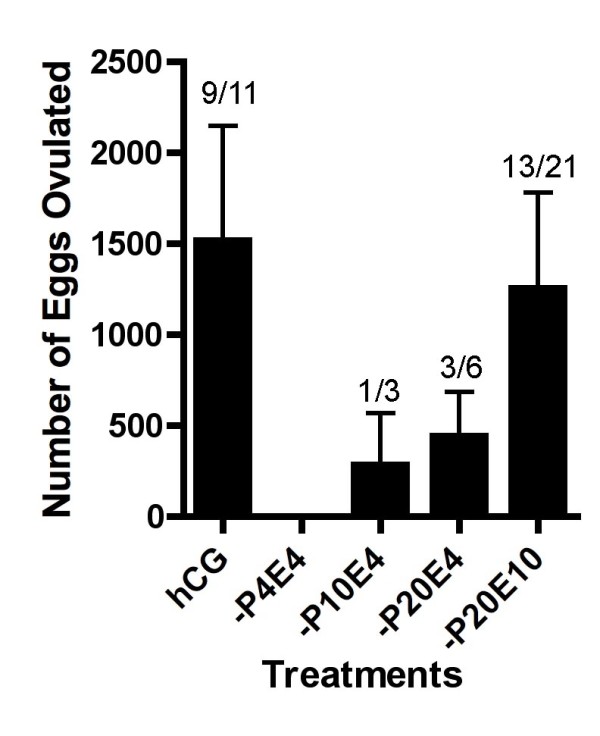
**Induction of ovulation by a mixture of steroids**. Frogs were treated with a mixture of estradiol (E) and progesterone (P) at the indicated concentration. The left column represents induction results in response to PMSG-hCG injection. After 24 to 30 hr incubation, the numbers of ovulated eggs were counted as described in the Materials and Methods. Each value represents the mean of data from more than three different females. The number of ovulated frogs per number treated is indicated over each column. Vertical lines indicate standard deviation.

**Figure 4:**
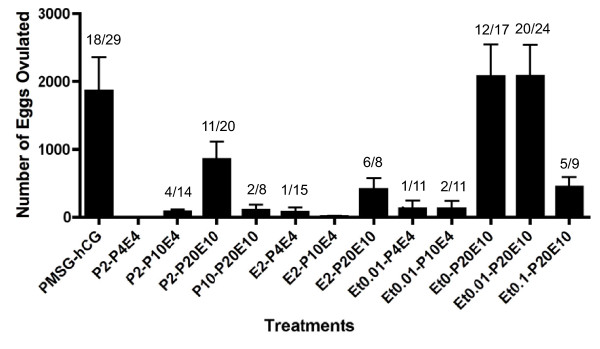
**Synergistic effects of pre-treatment and a cocktail of steroids upon induction of ovulation**. Frogs were pre-treated with or without (Ethanol alone) steroids, or at the indicated concentrations, for one day before ovulation induction by a mixture of steroids. Frogs were then treated with a mixture of estradiol and progesterone at the indicated concentrations. After 24 to 30 hr incubation, the numbers of ovulated eggs were counted as described in the Materials and Methods. Annotations underneath each column indicate substance and concentration for both pre-treatment and the induction of ovulation. For example, Et0-P20E10 represents pre-treatment with 0% ethanol for one day followed by induction by a mixture of 20 μM progesterone and 10 μM estradiol the following day. The left column represents the induction results in response to PMSG-hCG injection. Each value represents the mean of data from more than three different females. The number of ovulated frogs per number treated is indicated over each column. Vertical lines indicate standard deviation.

To evaluate the effect of steroids upon frog ovulation, we applied steroids directly into the water in which *Xenopus *were being maintained. Of the steroids examined, progesterone was shown to induce ovulation *in vivo *but only if applied at high concentration (Figure 1). However the frequency of frogs successfully ovulating and the number of eggs ovulated by progesterone alone was significantly lower than when ovulation was induced by hCG-injection. We next investigated the effect of pre-treatment or applying a mixture of steroid hormones. When frogs were pre-treated with low concentrations of steroids, the frequency of frogs in which ovulation was induced was reduced (Figure 2). Estradiol exhibited a potent inhibitory effect upon progesterone-induced ovulation during pre-treatment. However¸ we observed a synergistic effect of estradiol and progesterone in the induction of ovulation by adding a mixture of these steroids (Figure 3). When the concentration of estradiol mixed with progesterone was increased, we observed an increase in the number of eggs ovulated. We then demonstrated the effect of pre-treatment and using treatments composed of mixed steroids. Our results confirmed that we had discovered a novel method for the induction of ovulation in *Xenopus *that did not use injection needles but caused ovulation in a similar number of animals as PMSG-hCG injection protocols. Moreover, there was no significant different in the number of oocytes released when compared to standard injection protocols (Figure 4). As shown in Figure 4, thousands of oocytes were released in response to pre-treatment with 0.1 M NaCl alone, or with 0.01% ethanol, followed by exposure to a mixture of 20 μM progesterone and 10 μM estradiol (Et0-P20E10 and Et0.01-P20E10).

**Figure 5:**
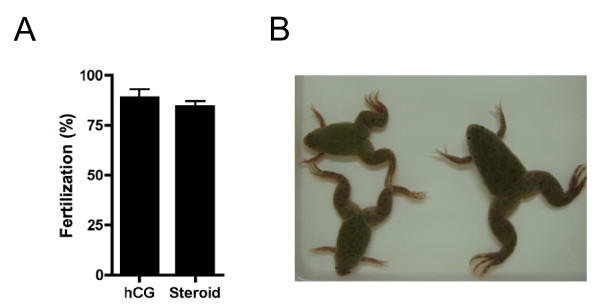
**Externally applied steroids induced natural spawning**. (A) Artificial insemination was conducted for eggs squeezed during spawning induced by a mixture of steroids (Steroid). As a control, the fertilization rate of eggs ovulated by PMSG-hCG injection was examined (hCG). Fertilisation rate (in %) was assessed by counting the number of eggs dividing to the 4 cell stage following insemination. Each value represents the mean of data from three different females. Vertical lines indicate standard deviation. (B) A photograph of frogs developed from eggs ovulated by steroids.

In order to demonstrate that eggs ovulated in response to steroid induction protocols were normal, we conducted *in vitro *insemination assessments. Data revealed that eggs ovulated in response to steroid treatment possessed identical ability to fertilize as those ovulated by standard hCG-injection protocols (Figure 5A). Eggs fertilized *in vitro *developed normally (Figure 5B). Results strongly indicated that steroid-induced ovulation was identical to physiological ovulation.

## Discussion

In the present study we demonstrated that ovulation could be induced in frogs by simply adding steroids into the water, a method previously shown to be efficient for the induction of ovulation in teleost fish. Our new method is relatively simple, and more importantly, avoids the need for needle injection. As the concentration of steroids in the stock solution is extremely high (200 mM for progesterone and 100 mM for estradiol), these chemicals are normally crystallized when kept at 4°C. However, these stock solutions can be kept at room temperature for up to six months in order to avoid crystallization. To investigate how much amount of steroids incorporated into frog tissues in the treatment established in this study, we conducted biochemical determination of incorporation of steroids into tissues of frogs using ^3^H tracers of progesterone and estradiol. The result is shown in Table.1. Although only 1% of added steroids were dissolved into water, ten-times higher concentration of progesterone was detected in ovary after 24 hr treatment. The result demonstrated that there are some mechanisms to accumulate progesterone into ovary and by the mechanisms concentration of progesterone in the ovary became high enough to induce oocyte maturation and ovulation. The results also indicated that the reason why extremely high concentration of steroids is necessary for induction of ovulation.

The fertilization rate of eggs obtained by the new induction method was comparable to those observed with eggs ovulated by standard hCG-injection methods. Moreover, juveniles developed normally. Females induced to ovulate by the new method appeared healthy and no damage was observed following treatment. Importantly, females could be re-used in further induction experiments 3-4 months after initial treatment. This is in line with timescales recommended for hCG-injection treatment in frogs. Consequently, we have successfully described a new method for the induction of frog ovulation without injection. Oocytes in *Xenopus laevis *ovary were classified into 6 stages [10]. hCG injection releases only the oocytes in stage VI. The effects of mixtures of steroids over a day time period as used in this study may have stimulus effect on oocyte growth in one or more of the stages. In fact we could induce ovulation only a month after previous treatment with steroids (Data not shown).

However, basic questions were raised during the course of our study, which should be given due consideration. Firstly, we need to address the stimulatory effect of estradiol upon the induction of ovulation. Secondly, we need to address the stimulatory effect of pre-treating frogs with 0.1 M NaCl. These concerns are discussed below.

The effect of estradiol upon ovulation has been studied in several different types of frog. It has also been established that pituitary extracts (PE) exhibit ovulating-inducing activity, both *in vivo *and *in vitro*. In frogs, ovulation can be induced *in vitro *in dissected ovarian pieces (ovarian fragments) by adding PE into culture medium. Although the effects of steroids were only studied in conjunction with pituitary extracts *in vivo*, it was demonstrated that steroids alone can induce ovulation by using ovarian fragments in *Xenopus *and *Rana pipiens *[11,12]. Of the steroids tested, progesterone was the most potent in terms of ovulation-inducing activity. Subsequently, Wright examined the effect of steroids upon pituitary extract-induced and progesterone-induced ovulation using ovarian fragments *in vitro *[13]. Estrogens exerted inhibitory effects upon pituitary extract-induced ovulation, but when combined with progesterone, or PE and progesterone, caused enhanced ovulation. In the present study, we demonstrated the stimulatory activity of estradiol upon progesterone-induced ovulation *in vivo*. However, pre-treatment with estradiol reduced the rate of ovulation, while estradiol also inhibited PE-induced ovulation *in vitro*. We propose that estrogens possess stimulatory-activity upon the process of ovulation but will reduce the competence of oocyte maturation if used in pre-treatments. Recently, Pang et al. used the zebrafish model to demonstrate the down-regulation of the membrane progestin receptor (mPR) responsible for progestin-induced oocyte maturation in response to estrogen acting via the membrane estrogen receptor [14]. In *Xenopus*, it has been demonstrated that the mPRβ subtype is a receptor for progesterone [4]. Thus, it can be speculated that pre-treatment with estradiol for 24 hours can induce the down-regulation of mPRβ prior to the induction of ovulation resulting in the inhibition of oocyte maturation and ovulation. On the contrary to accumulation of progesterone in ovary, the concentration of estradiol in the ovary after incubation was almost same as in the solution (Table 1). It is known that estradiol has bi-modal effects on gonadotropin synthesis in mammals [15]. It is possible that estradiol possesses similar effects in amphibians and stimulates the synthesis of gonadotropin. Increased amount of gonadotropin might show synergic effect with progesterone to induce ovulation.

**Table 1:** Incorporation of steroids into tissues of *Xenopus*

**Steroids**		**Progesterone**			**Estradiol**	
Added ^3^H-steroid (dpm/g)		477,700 ± 8,190			293,600 ± 39,240	
Dissolved in 0.1 M NaCl (dpm/g)		3,943 ± 438			3,777 ± 681	
Estimated concentration (μM)		0.17 ± 0.02			0.13 ± 0.02	
Tissues	Blood	Ovary	Muscle	Blood	Ovary	Muscle
Incorporation into tissues (dpm/g)	17,180 ± 557	38,380 ± 5,252	14,920 ± 1,670	4,671 ± 365	4,280 ± 1,080	2,236 ± 328
Estimated concentration (μM)	0.72 ± 0.02	1.61 ± 0.22	0.62 ± 0.07	0.16 ± 0.01	0.15 ± 0.04	0.08 ± 0.01

Each value in the mean (± S.D.) of three separate experiments using samples of three separate bottles or females

The stimulatory effect of pre-treatment with a mere salt solution is also surprising. The jelly layer surrounding ovulated *Xenopus *eggs is sticky in nature and tends to adhere to vessels. To avoid the absorption of eggs onto vessels, salt solution is normally applied to the water used to house frogs during ovulation. For these reasons, we also used salt water to collect and count the number of eggs. Surprisingly, pre-treatment with this solution alone caused a significant increase in the number of eggs ovulated. Generally, it is recommended to pass water activated charcoal to remove chlorine prior to exposure to frogs. However, there are no published reports of studies investigating the effect of chlorine upon ovulation in *Xenopus*. For the present study, we used ultra-pure water to prepare 0.1 M NaCl. We consider that the pre-treatment of frogs with water from which all traces of chlorine had been removed, caused an increase in the competency of oocytes to undergo maturation. Alternatively, it is possible that a hypertonic solution could cause physiological changes to frogs that may inadvertently induce ovulation. We were unable to find reports in the current scientific literature that connect high salt conditions with the induction of ovulation. However, abrupt changes in osmotic pressure could manifest in physiological changes. The precise reason for the induction of ovulation by salt solution remains to be addressed and should form the focus for further study.

## Conclusions

We established an efficient means of inducing ovulation in *Xenopus laevis *which involved pre-treatment of frogs with salt solution followed by a mixture of estradiol and progesterone.

## Competing interests

The authors declare that they have no competing interests.

## Authors' contributions

AO, JD and YI carried out assay for ovulation. TT participated in the design of the study and drafted the manuscript. All authors read and approved the final manuscript.
